# Appeal for Aid

**Published:** 1900-01

**Authors:** 


### Abstracts and Selections.

### Appeal for Aid.

The following letter has been forwarded by the Woman's Committee of the Psychological Section of the Medico-Legal Society, in response to an appeal for aid in fitting out a Hospital Ship for South African wounded soldiers.

Chairman.—Prof. W. Xavier Sudduth, Chicago, Ill.

Legal and Scientific. Vice-Chairmen.—Clark Bell, Esq., New York; Rev. Antoinette B. Blackwell, New York; Harold Browett, Esq., Shanghai, China; C. Van D. Chenoweth, Worcester, Mass.; Judge Abram H. Dailey, Brooklyn; Moritz Ellinger, Esq., New York; Rev. Phoebe A. Hanaford, New York; Thompson Jay Hudson, Esq., Washington, D. C.; Sophia McClelland, New York; Mrs. Jacob F. Miller, New York.

Medical. Vice-Chairmen.—James R. Cocke, M. D., Boston, Mass.; T. D. Crothers, M. D., Hartford, Conn.; F. E. Daniel, M. D., Austin, Texas; H. S. Drayton, M. D., New York; Wm. Lee Howard, M. D., Baltimore, Md.; Henry, Hulst, M. D., Grand Rapids, Mich.; Prof. Thomas Bassett Keyes, Chicago, Ill.; R. J. Nunn, M. D., Savannah, Ga.; A. E. Osborne, M. D., Glen Ellen, Cal.; Jas. T. Searcy, M. D., Tuscaloosa, Ala.; U. O. B. Wingate, M. D., Milwaukee, Wis.; Mrs. Mary Louise Thomas, New York.

Secretary and Treasurer.—Clark Bell, Esq., New York.

Executive Committee.—Clark Bell, Esq., Chairman; Caroline J. Taylor, Secretary; M. Ellinger, Esq.; S. B. W. McLeod, M. D.; Judge A. H. Dailey; Ida Trafford Bell; Geo. W. Grover, M. D.; H. W. Mitchell, M. D.

New York, November 6, 1899.

*Lady Randolph Churchill.*

Honored Madam: The undersigned Woman's Committee of the Psychological Section of the Medico-Legal Society note with pride and pleasure your praiseworthy efforts to furnish and despatch a Hospital Ship for the relief of the wounded soldiers and sailors of the Nation, of which you have become a citizen, by your marriage to a subject of the English throne; and we have heard your appeal to the women of the country of your birth, to aid you in a work of philanthropy and charity for the benefit of the soldiers and sailors of the country of your adoption, in the unfortunate war now going on in the South African Republic.

We beg to remind you that there is now an Amercian lady languishing in an English prison, who is universally believed by her countrymen and countrywomen, to be entirely innocent of the crime with which she is charged; who was sentenced to death; whose sentence was commuted, on the advice of the judge who tried her, by the English Home Secretary, on the ground that a doubt existed as to whether the death of her husband was caused by, or due to, arsenic; and who has, as we believe, suffered an unjust imprisonment for more than ten years. That this is despite the petitions and appeals of thousands of her countrymen and countrywomen; of the strong and urgent appeal of the Lord Chief Justice of England, who believed her innocent; of the personal urgent appeal of President Benjamin Harrison and the members of his Cabinet; of the strong letters of the wife of the President, and the views of the American Cabinet Ministers, appealing to the Queen of England in her behalf; all of whom urged her release, because they believed her to be innocent; and whom the Hon. William McKinley, President of the United States, with a firm conviction of her innocence, has asked for her release from the English government, as an act of international comity, between two great nations, whose people are now anxious to see a strong feeling of amity cemented between these nations of a common blood and origin.

We ask you to seize the present occasion to urge upon the Home Secretary for England the release of this American lady, who, less fortunate than yourself in her marriage with an Englishman, has since his death stoutly claimed her American citizenship, as she has the legal right to do, under the English law, and which she never surrendered under the American law; who is now suffering a terrible, cruel and long continued imprisonment, longer than any woman has endured under the English law, as we believe, and longer than any English woman of a similar social position would have suffered, if believed by the English authorities to be guilty; who, reared as a lady, and the daughter of one of our best families in America, in her feeble and delicate health, has maintained and preserved the rank of a star prisoner by her good behavior.

We urge upon you to explain to the English Home Secretary that his refusal to recommend Florence E. Maybrick to the clemency of the Queen of England, now so earnestly desired by the American President and people; so stoutly and constantly urged by the American Ambassador, Hon. Joseph H. Choate; is not only a source of constant irritation to our people in America, but that it disturbs and prevents that sincere friendship, which has been made more necessary by the progress of recent events, involving the interests of both countries, growing out of the recent war between America and Spain, and the present conflict in South Africa; and that it also stands in the way of our coöperation with you in your humane endeavors, because of a feeling of just resentment and extreme annoyance at the continuance of a punishment, against an American citizen, whom we believe innocent of the offense for which she has been tried, that is more terrible and severe to endure than was that of Dreyfus.

Aid us, dear Lady Churchill, in securing the release of this innocent lady, and we will pledge the women of America to double the effort you are now making for the wounded in South Africa.

We remain,

Very faithfully yours,

Caroline J. Taylor, Chairman,
M. Louise Thomas,
The Countess Bettini di Moise,
Florence Dangerfield Potter,
Rosalie Dailey,
Phoebe A. Hanaford,
Ida Trafford Bell,
Sophia McClelland,
Antoinette Brown Blackwell,
C. Van D. Chenoweth,

Composing the Woman's Committee of the Psychological Section of the Medico-Legal Society of New York.

—*Advance Sheets Medico-Legal Journal.*

